# Hybrid Polyethylene Glycol/Sodium Metaphosphate Composites Prepared via Coacervation

**DOI:** 10.3390/nano12030528

**Published:** 2022-02-03

**Authors:** Bruno Poletto Rodrigues, Guilherme Nunes Braga Maurício de Macedo, Yang Xia, Andrea Balducci, Lothar Wondraczek

**Affiliations:** 1 Otto Schott Institute of Materials Research, Friedrich Schiller University Jena, 07743 Jena, Germany; guilherme.bnmm@uni-jena.de (G.N.B.M.d.M.); yang.xia@uni-jena.de (Y.X.); lothar.wondraczek@uni-jena.de (L.W.); 2 Institute of Technical Chemistry and Environmental Chemistry, Friedrich Schiller University Jena, 07743 Jena, Germany; andrea.balducci@uni-jena.de; 3 Center of Energy and Environmental Chemistry, Friedrich Schiller University Jena, 07743 Jena, Germany

**Keywords:** ionic conduction, phase separation, glasses, composite materials, 66.10.Ed, 64.75.+g, 61.43.Fs, 72.80.Tm

## Abstract

We report on the fabrication and characterization of homogeneous, monophasic sodium metaphosphate and polyethylene glycol hybrid composites achieved via coacervation in aqueous solution. After separation and drying, an amorphous plastic solid is formed, composed mostly of hydrated sodium phosphate moieties amalgamated with polyethylene glycol chains. These composites are largely X-ray amorphous and can contain up to 8 weight percent of polymer. Impedance spectroscopic measurements reveal DC conductivity values of 12 μS/m at room temperature, an enhancement of three orders of magnitude when compared to glassy sodium metaphosphate, and the presence of the polyethylene glycol is reflected in the equivalent circuit and ionic hopping analyses.

## 1. Introduction

Composite polymer–ceramic materials are widely studied options for the realization of solid-state electrolytes for lithium [1,2,3] and sodium ion batteries [4,5], and they are typically prepared via slurry casting or spraying. While these processes allow for high compositional flexibility, achieving the final, multiphasic composites requires the evaporation of organic solvents or in situ polymerization [6]. Alternatively, coacervation is a type of liquid–liquid phase separation that can happen in polymer solutions, producing a more dense phase, the *coacervate*, and a less dense phase, the *supernatant* [7]. The coacervation process in aqueous solutions is traditionally categorized either as *simple coacervation*, where the phase separation is triggered by the addition of a desolvation agent (also called coacervation or inducing agent) which changes the solubility equilibrium of the system, or as *complex coacervation*, where the electrostatic forces between charged molecules and polymers determine the phase separation behavior [8,9]. Pioneering studies on the coacervation of alkali phosphate aqueous solutions utilized the addition of alkaline earth halides to trigger the phase separation [10,11] where the electrostatic interactions between the polyphosphate chains and the polyvalent cations induced the coacervate formation [12,13,14,15,16]. The addition of polar organic solvents such as methanol [10] and ethanol [17] has also been observed to provoke coacervation in phosphate aqueous solutions. This behavior has been hypothesized to be based on the decrease in the solution’s static dielectric constant with increasing organic solvent concentration, leading to a decreased solubility of the phosphate molecules [18], similar to the so-called salting-out effect observed in ternary water–polymer–electrolyte solutions [19]. More recently, several authors have used pyrophosphate coacervates precipitated from aqueous solutions by the addition of ethanol as precursor materials in the fabrication of glasses for optical and biological applications [18,20,21,22]. Likewise, aqueous solutions of polyethylene glycol (PEG) usually display immiscibily gaps [23] and this behavior is also observed in water–PEG–salt solutions [24,25]; such aqueous biphasic systems—with the addition of salts—have been widely studied and found many biological and pharmaceutical applications [26,27,28]. In this paper, we report on the on hybridization of sodium metaphosphate glasses with PEG via combination of precursor aqueous solutions, coacervation, and controlled drying of the coacervate, enabling the preparation of homogeneous organic–inorganic composites which cannot be formed via traditional melt-quenching or slurry processing techniques.

## 2. Materials and Methods

### 2.1. Coacervate Formation

Precursor solutions of $4\;\mathrm{mol}\;L^{-1}$ ${\mathrm{NaPO}}_{3}$ (NP) were prepared by mixing the appropriate proportions of deionized water and ${\mathrm{NaPO}}_{3}$ glass powder (synthesis method previously described [29]). Liquid coacervation agents (ethanol (99.8%, Carl Roth GmbH, Karlsruhe, Germany), ethylene glycol (VEB Laborchemie Apolda, Apolda, Germany—EG), and polyethylene glycol (average $M_{n}$ = 190–210 $\;g\;{\mathrm{mol}}^{-1}$, Sigma-Aldrich 807483, Merck KGaA, Darmstadt, Germany—PEG200) were gradually added to the parent NP solution until it turned from transparent to milky, and the added volume noted. Then, water was added to the milky solution until it turned clear again, the volume noted and the procedure repeated several times.

The static dielectric constant $\varepsilon_{0}$ of the solutions was estimated using Oster’s rule for the polarization of fluid mixtures $p_{mix}$,

(1) $$p_{mix}=\frac{\sum_{i=1}^{n}x_{i}v_{i}p_{i}}{\sum_{i=1}^{n}x_{i}v_{i}},$$

where $x_{i}$, $v_{i}$, and $p_{i}$ are the molar fraction, molar volume, and polarization of each component *i*, respectively. This quantity is directly related to $\varepsilon_{0}$ by

(2) $$p=\frac{\left(\varepsilon_{0}-1\right)\left(2\varepsilon_{0}+1\right)}{9\varepsilon_{0}}.$$

For the coacervation agents and water, the polarization was calculated directly from literature values of the dielectric constant. For the sodium metaphosphate solution, its $\varepsilon_{0}$ was estimated with

(3) $$\varepsilon_{0}=\frac{\varepsilon_{0,sol}}{1+\sum_{i}A_{i}x_{i}\ln\left(1+B_{i}\sqrt{I_{X}}\right)},$$

where $\varepsilon_{0,sol}$ is the dielectric constant of the solvent (in this case, water), $I_{X}$ is the solution’s ionic strength, and $A_{i}$ and $B_{i}$ are constants for each of the *i* ions present [30]. Wang and Anderko report $A_{Na^{+}}=0.523741$ at 25 °C, and $B_{i}$ = 1,027,541 was found to give good results for a wide variety of ionic species in aqueous solution [30]. $A_{PO_{3}^{-}}=0.53496$ was estimated using published literature data [31]. Table 1 summarizes the data used to estimate the $\varepsilon_{0}$ of the sodium metaphosphate solution–water–coacervation agent ternary solutions following Equation (1), with the values for the polarization of each component estimated from their dielectric constant via Equation (2).

### 2.2. Dried Coacervate Sample Preparation

**Figure 1:**
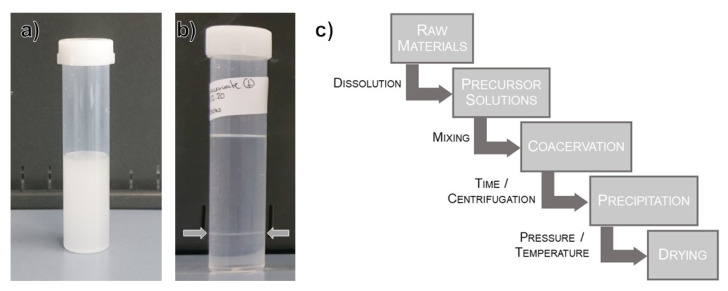
Outline of sample preparation: (**a**) Mixture of the precursor solutions shortly after the onset of coacervation; (**b**) Mixture of the precursor solutions after phase separation and precipitation for 24 h—the arrows show the boundary between the coacervate and the supernatant; and (**c**) Diagram of the dried coacervate preparation process.

Precursor solutions of $4\;\mathrm{mol}\;L^{-1}$ ${\mathrm{NaPO}}_{3}$ and $0.4\;\mathrm{mol}\;L^{-1}$ PEG1000 were prepared by mixing the appropriate proportions of deionized water, ${\mathrm{NaPO}}_{3}$ glass powders and polyethylene glycol (average $M_{n}$ = 950–1050 $\;g\;{\mathrm{mol}}^{-1}$, Sigma Aldrich P3515, Merck KGaA, Darmstadt, Germany—PEG1000). An aqueous solution of PEG1000 was chosen as coacervation agent for the dried samples to exacerbate the desolvation effect observed in the coacervate formation studies. A total of 32 mL of the PEG1000 solution was added to a beaker, and the ${\mathrm{NaPO}}_{3}$ solution was slowly combined under continuous stirring until the solution turned from clear to milky, which happened after the addition of 15 mL NaPO_3_ solution. This was taken as the onset of phase separation, and the solution was then allowed to settle for 24 h. After precipitation, 1 mL aliquots of the supernatant and the coacervate were pipetted out of the mother solution, put in small open sample holders, transferred to a vacuum oven, and dried for 67 h at 35 °C and 50 kPa absolute (Figure 1 summarizes the sample preparation process). Sample drying was followed with the pressure gauge of the vacuum oven; prior to 62 h, the vacuum pump had to be regularly activated to maintain the set pressure, then after confirming the vacuum was holding without further pumping for 5 h, the samples were considered “dry” and taken out of the oven. After drying, the supernatant turns into a transparent, fragile solid, while the coacervate becomes a translucent and considerably plastic solid.

### 2.3. Sample Characterization

Raman spectra of the dried coacervate and supernatant samples were measured with a Renishaw inVia Raman microscope at 20× magnification with an excitation wavelength of 633 nm over the range of 110 to 1325 cm^−1^. Each spectra is averaged over 20 measurements, and is corrected for baseline and thermal population [36] and normalized via standard normal variate (SNV) [37] procedure.

Simultaneous differential scanning calorimetry and thermogravimetric analysis coupled with mass spectroscopy (DSC-TGA coupled MS) measurements of the dried coacervate were performed with a Netzsch 449 F1 coupled to a Netzsch Aeolos 403D mass spectrometer. A total of 25 mg samples were put in aluminum crucibles, stabilized at 40 °C, and then heated to 550 °C with a heating rate of 10 °C min^−1^. The mass spectra were measured by a CH-Tron detector with 1100 V SEM voltage and mass window of 16 to 50 u.

X-ray diffraction patterns of pristine and heat-treated samples (heating rate of 10 °C min^−1^ up to 170, 300 and 550 °C) were measured with a bench-top Rigaku Miniflex 300/600, using a Cu-K*α* X-ray source at 40 kV and 15 mA, a D/teX Ultra silicon strip detector, scanning the range of 3 to 90° 2*θ* with a scan speed of 1° min^−1^ and step width of 0.02°.

Ionic conductivity was measured with square samples placed between steel electrodes in a Novocontrol Alpha-A Analyser with a Novotherm Temperature Control System in the frequency range of 0.1 Hz to 10 MHz and temperatures between 25 and 50 °C in 5 °C steps. The real part of conductivity $\sigma^{\prime }$ is calculated from complex impedance as $\sigma^{\prime }=Z^{\prime }/\left({Z\prime }^{2}+{Z\prime \prime }^{2}\right)\cdot \left(l/A\right)$, where $Z^{\prime }$ and $Z^{\prime \prime }$ are the real and imaginary part of complex impedance, *l* is the sample thickness and *A* is the sample area. The impedance response of each sample was modeled with a parallel R-CPE equivalent circuit, with the following fitting parameters: the resistance R, the frequency-independent CPE parameter Q and the constant phase 0≤α≤1 [38]. The crossover frequency $f^{*}$ between the DC and AC conductivity regimes is defined as the frequency that fulfills the relation $\sigma^{\prime }\left(f^{*}\right)=2\sigma_{\mathrm{DC}}$ [39].

## 3. Results

### 3.1. Phase Separation and Coacervate Formation

**Figure 2:**
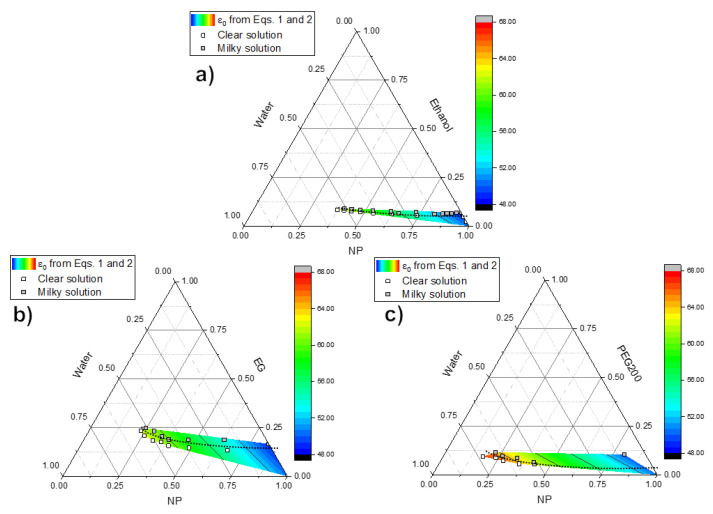
Ternary volume fraction graphs showing the estimated phase separation boundary (as a dashed line) for the sodium metaphosphate precursor solution with addition of water and (**a**) ethanol, (**b**) ethylene glycol, and (**c**) PEG 200.

Figure 2 summarizes our phase separation experiments in the estimated phase diagrams for the NP–water–(ethanol, ethylene glycol, and polyethylene glycol 200) systems with spinodal decomposition boundary and values for the solution’s static dielectric constant calculated from Equation (1). The phase separation boundary was found to be practically parallel to the NP axis in the ternary graphs (see Figure 2), while the dielectric constant strongly increases with increasing water content. Starting with the NP solution, the addition of any coacervation agent decreases the solution’s dielectric constant and triggers the phase separation, in line with previously reported results [18]. The subsequent addition of water reverts the phase separation and greatly increases solution’s the dielectric constant. Further rounds of alternated coacervation agent and water additions replicate the pattern of triggering and reverting the phase separation, but the values of the dielectric constant never reach the same lower limit achieved after the first coacervation agent addition. As more water is required to revert the phase separation than coacervation agent is needed to trigger it, a steady increase in $\varepsilon_{0}$ is observed along the phase separation boundary, and no critical threshold value of the solution’s static dielectric constant could be determined.

### 3.2. Raman Spectra

**Figure 3:**
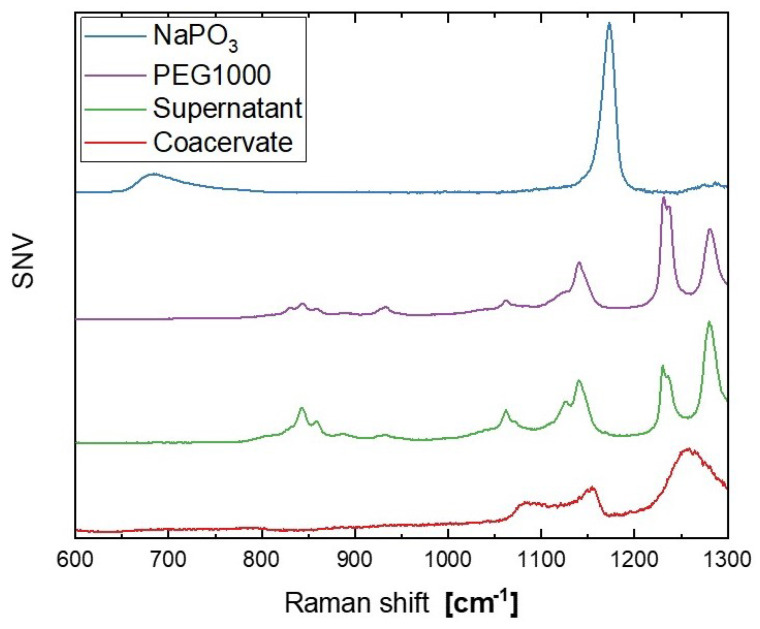
Raman spectra of the dried coacervate and supernatant from the starting glassy ${\mathrm{NaPO}}_{3}$ and PEG1000. The spectra are shifted vertically for improved readability.

A comparison between the Raman spectra of the original sodium metaphosphate ${\mathrm{NaPO}}_{3}$ glass, neat PEG1000, and the resulting dry supernatant and coacervate is shown in Figure 3. The ${\mathrm{NaPO}}_{3}$ spectra exhibits the expected strong features at approximately $680\;{\mathrm{cm}}^{-1}$ and $1170\;{\mathrm{cm}}^{-1}$, associated with the ${\left(P-O-P\right)}_{\mathrm{sym}}$ and ${\left({\mathrm{PO}}_{2}\right)}_{\mathrm{sym}}$ vibrations, and the broader, weaker signal at approximately $1280\;{\mathrm{cm}}^{-1}$ of the ${\left({\mathrm{PO}}_{2}\right)}_{\mathrm{asym}}$ stretching modes of the $Q^{2}$ species (where n in $Q^{n}$ denotes the number of bridging oxygen species in a $\left({\mathrm{PO}}_{4}\right)$ tetrahedra) [40]. The Raman spectrum of PEG1000 contains three band envelopes: two related to different motions of the ${\mathrm{CH}}_{2}$ species (rocking between 800 and $950\;{\mathrm{cm}}^{-1}$ and twisting between 1220 and $1300\;{\mathrm{cm}}^{-1}$) and one related to the C-O,C-C stretching at $1130\;{\mathrm{cm}}^{-1}$ [41]. The Raman spectra of the dried supernatant essentially mirrors the original PEG1000 spectra, with the changing peak intensities likely related to shifting chain configurations [41,42]. On the other hand, the dried coacervate shows only two features: a broad envelope between two weak peaks at 1080 and $1150\;{\mathrm{cm}}^{-1}$ and a broad peak centered at $1260\;{\mathrm{cm}}^{-1}$.

### 3.3. Thermal Analyses

**Figure 4:**
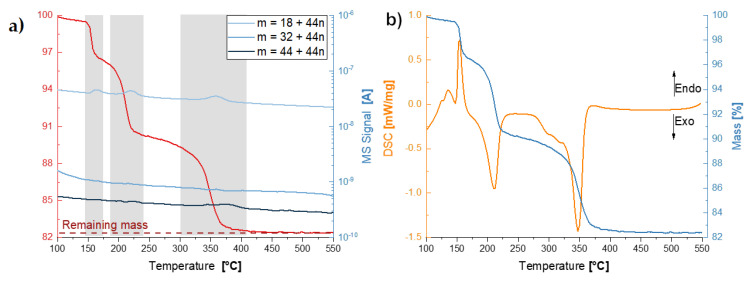
Thermogravimetric coupled with mass spectroscopy and differential scanning calorimetry scans of the dried coacervate: (**a**) TGA with coupled MS curves; and (**b**) DSC and TGA curves.

**Figure 5:**
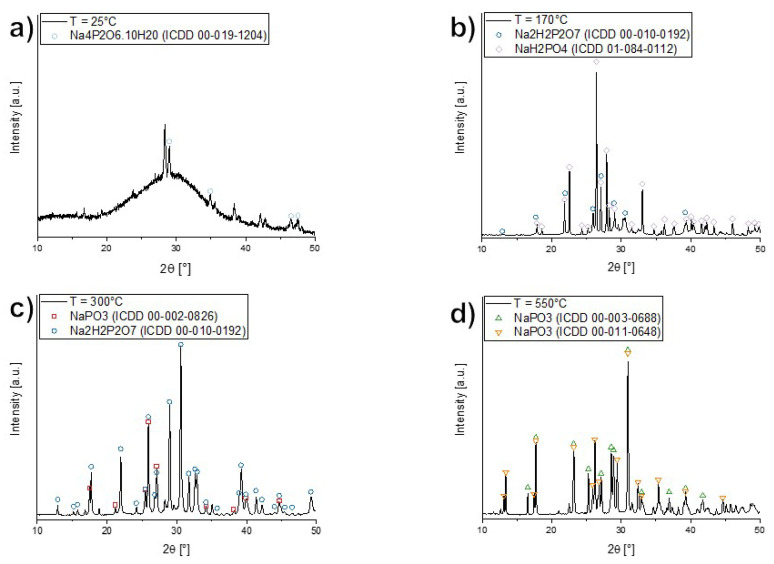
X-ray diffraction patterns for dried coacervate at different temperatures and associated crystalline phases and database card numbers: (**a**) Room temperature; (**b**) 170 °C heat treatment; (**c**) 300 °C heat treatment; and (**d**) 550 °C heat treatment.

Figure 4 depicts the thermogravimetric coupled with mass spectroscopy and differential scanning calorimetry scans of the dried coacervate in the temperature range of 100 to 550 °C. The TGA shows three distinct weight loss events roughly at 160, 210, and 350 °C and the coupled MS signals for the channels corresponding to water (18Da), and the main series of polytheylene glycol pyrolyzates in inert atmospheres: dihydroxyl (18+44nDa), methyl ether (32+44nDa) and vinyl ether (44+44nDa) [43]. The DSC scan shows that the first weight loss event at 160 °C coincides with an endothermic peak, while the two other weight loss events are associated with exothermic peaks. The weight loss stabilizes at approximately 18% above 400 °C. The TGA results show the dried coacervate to be composed of approximately 82 wt.% sodium phosphate, with water content between 10 and 18 wt.% and PEG content between 0 and 8 wt.% (Figure 4). The weight loss events at 160, 210 °C are due to water following the MS signal for 18 Da, and the 350 °C weight loss is a combination of further water evolution and the pyrolysis of PEG [44,45]—the MS signals for water and the dihydroxyl pyrolizates overlap. This series of water loss events is corroborated by the X-ray diffraction experiments (Figure 5), where the progression from highly hydrated phosphates to anhydrous but depolymerized phosphates and to fully polymerized phosphates is clearly observed. Our results are consistent with the dehydration of sodium phosphates as reported by de Jager and Prinsloo [46]. From X-ray diffraction patterns taken from pristine and heat-treated samples at 170, 300, and 550 °C (after each DSC peak), the following structural evolution is observed (see Figure 5): the dried coacervate is predominantly X-ray amorphous while also having some crystalline peaks characteristic of heavily hydrated phosphate ${\mathrm{Na}}_{4}P_{2}O_{6}.10H_{2}O$, after the first weight loss event accompanied by the endothermic DSC peak, the amorphous halo and the hydrated phosphate disappear while reflexes from sodium dihydrogen phosphate ${\mathrm{NaH}}_{2}{\mathrm{PO}}_{4}$ and disodium dihydrogen diphosphate ${\mathrm{Na}}_{2}H_{2}P_{2}O_{7}$ (anhydrous phases with structural water) appear; at 300 °C, after the second weight loss event and the first exothermic peak, all sodium dihydrogen phosphate has disappeared while the disodium dihydrogen diphosphate begin to convert to sodium metaphosphate, losing its structural water; and finally, at 550 °C, only crystalline sodium metaphosphate remains.

### 3.4. Complex Impedance Spectroscopy

**Figure 6:**
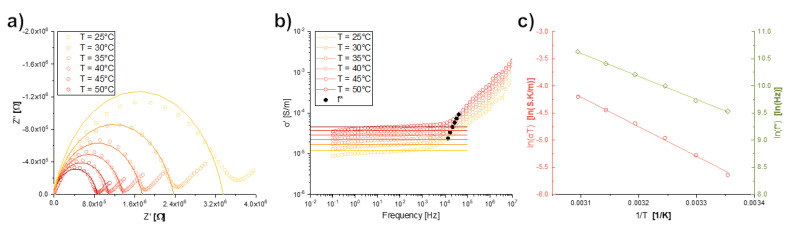
Results from complex impedance characterization of the dried coacervate from 25 to 50 °C: (**a**) Nyquist plot showing the measured impedance and the fitted R-CPE equivalent circuits in solid lines; (**b**) Bode plot with calculated DC conductivity from the R-CPE circuits as solid lines and the DC to AC crossover frequencies; and (**c**) Arrhenius plot of the DC conductivity and crossover frequency. The error bars are smaller than the data point size. Solid lines represent linear fits to the data.

Figure 6 shows the experimental complex impedance data for the dried coacervate. The fitting parameters of the best-fit parallel R-CPE equivalent circuits shown in the Nyquist plot are summarized in Table 2 together with the circuit’s calculated DC conductivity and crossover frequency. The Bode plot shows good agreement between the experimental conductivity with the DC conductivity from the equivalent circuit. The AC to DC crossover frequency $f^{*}$ is also shown, which is related to the ionic hopping frequency [47]. From the DC conductivity’s Arrhenius plot, a conductivity activation energy of 46±1 kJ/mol and pre-exponential term $\sigma_{0}=\left(400\pm 200\right)$ kS/m are determined. Additionally, an activation energy of 35.3±0.6 kJ/mol and pre-exponential term $f_{0}=\left(21\pm 5\right)$ GHz are found for the crossover frequency.

## 4. Discussion

Our initial results from the phase separation behavior of our studied system, summarized in Figure 2, seem to indicate that the mechanism of coacervation of sodium metaphosphates is intermediate between the simple and complex coacervation cases. As in complex coacervation, the inter-molecular electrostatic forces play an important role [17,18] as the phase separation boundary lies between a high and low dielectric constant values. However, the addition of water causes both the reversal of the phase separation and a strong increase in $\varepsilon_{0}$, thus no singular threshold value of the dielectric constant could be assigned to trigger the phase separation. This can be interpreted as the effect of differential solubility as in simple coacervation, where the addition of the coacervation agent effectively “pulls” water from the around the sodium and phosphate ions, causing them to rebind (which is facilitated due to the lower dielectric constant of the solution) and precipitate. This is consistent with studies on the hydration of ethanol (2 to 3 water molecules) [48] and polyethylene glycols (4 to 6 water molecules per monomer for molecules above 1500 Da, down to a minimum of 2 per monomer with decreasing molecular weight) [49,50,51,52].

The addition of PEG1000 to the phosphate network via coacervation is reflected in the Raman spectra of the dried coacervate (Figure 3), which does not display the characteristic peaks of either component but shows great similarity to the Raman spectra of molten PEG1000 [42]. Due to differing Raman scattering cross-sections [53,54], the organic polymer contribution seems to overshadow the inorganic phosphate signals. This resulted in an increase in ionic conductivity of three orders of magnitude at room temperature when compared to glassy NaPO_3_ (12 μS/m versus 45 nS/m) [29], with the coacervate conductivity even approaching the reported values of polyethylene oxide–sodium ionic liquid mixtures (approximately 1 mS/m) [55]. The effect of water and polyethylene glycol in the sodium metaphosphate structure is apparent in the ionic hopping frequencies: for diffusive motion, the base hopping attempt frequency is expected to be equal to the atomic vibrational frequency $f_{0}$ [56], but in the coacervate the base attempt frequency is 21 GHz, much lower than the 6.4 THz measured for sodium ions in glassy sodium metaphosphate [57]. When the ionic motion is controlled by relatively large energetic barriers separating clusters of low-energy sites, the difference in timescales between inter- and intra-cluster hopping has to be taken into account as an entropic term which decreases the effective hopping frequency [58]. Following Garcia-Belmonte and Bisquert’s approach [56], the cluster size in the dried coacervates is estimated to be 33 nm, which is roughly the length of four PEG1000 molecules (polymerization degree of approximately 23 and 0.35 nm per monomer [59]). The presence of clusters is also supported by the depressed semicircles seen in the Nyquist plots (Figure 6), as divergence from ideal RC equivalent circuit behavior (α≈0.8, equivalent to a 72° phase angle) may originate from correlated back–forward ionic jumps [60] and the resulting distribution of diffusion timescales [38,61]. The energy barriers between clusters are also much lower in the coacervate than in glassy ${\mathrm{NaPO}}_{3}$ (35 and 70 kJ/mol, respectively), as the elastic moduli of the coacervate are much lower than the glass, therefore the inter-cluster ion hopping requires less elastic energy to be expended to deform the glassy matrix [62,63].

## 5. Conclusions

In this paper, we report on a novel method for preparing homogeneous organic–inorganic composites using coacervation. From aqueous solutions of sodium metaphosphate and polyethylene glycol, a coacervate was formed, dried, and characterized. The samples are composed mostly of sodium phosphate, with water and PEG as secondary components. The coacervates are predominantly X-ray amorphous after drying and recrystallize sodium metaphosphate when heat-treated. The room-temperature ionic conductivity of sodium metaphosphate is greatly enhanced via coacervation, due to the presence of low-activation-energy clusters around the polyethylene glycol chains.

## Figures and Tables

**Table 1:** Parameters used in the calculation of the static dielectric constants.

	ρ (g/${\mathrm{cm}}^{3}$)	$V_{M}$ (${\mathrm{cm}}^{3}/\mathrm{mol}$)	$\varepsilon_{0}$	*p*
NP	1.20±0.01	19.72	50.4 £	11.1
Water	0.997 †	18.02	80.1 †	17.69
Ethanol	0.789 †	58.39	25.3 †	5.51
EG	1.106 ‡	56.11	41.2 ¶	9.04
PEG200	1.120 §	178.57	22.1 ¶	4.79

£ Calculated with Equation (3); † Reference [32]; ‡ Reference [33]; ¶ Reference [34]; § Reference [35].

**Table 2:** Parameters of the R-CPE equivalent circuits (Resistance R, CPE constant Q, phase α) fitted to the experimental impedance data, circuit DC conductivity $\sigma_{\mathrm{DC}}$, and crossover frequency $f^{*}$ as a function of temperature.

Temperature (°C)	R (MΩ)	Q (p$S.s^{\alpha }$)	α	$\sigma_{\mathrm{DC}}$ (μS/m)	$f^{*}$ (kHz)
25	3.4 ± 0.5	60 ± 10	0.82 ± 0.01	11.90 ± 0.05	13.8
30	2.4 ± 0.3	100 ± 10	0.80 ± 0.01	16.83 ± 0.06	16.8
35	1.8 ± 0.3	110 ± 10	0.797 ± 0.009	22.82 ± 0.09	22.0
40	1.4 ± 0.2	120 ± 10	0.797 ± 0.007	29.2 ± 0.1	27.1
45	1.1 ± 0.2	140 ± 10	0.799 ± 0.006	36.9 ± 0.1	33.1
50	0.9 ± 0.1	140 ± 10	0.804 ± 0.006	46.4 ± 0.1	41.1

## Data Availability

The data that support the findings of this study are available from the corresponding author upon reasonable request.

## References

[1] Bu J., Leung P., Huang C., Lee S.H., Grant P.S. (2019). Co-spray printing of LiFePO_4_ and PEO-Li_1.5_Al_0.5_Ge_1.5_(PO_4_)_3_ hybrid electrodes for all-solid-state Li-ion battery applications. J. Mater. Chem. A.

[2] Li X., Cheng S., Zheng Y., Li C.Y. (2019). Morphology control in semicrystalline solid polymer electrolytes for lithium batteries. Mol. Syst. Des. Eng..

[3] Yu X., Manthiram A. (2020). A Long Cycle Life, All-Solid-State Lithium Battery with a Ceramic–Polymer Composite Electrolyte. ACS Appl. Energy Mater..

[4] Hou W., Guo X., Shen X., Amine K., Yu H., Lu J. (2018). Solid electrolytes and interfaces in all-solid-state sodium batteries: Progress and perspective. Nano Energy.

[5] Yu X., Xue L., Goodenough J.B., Manthiram A. (2020). All-Solid-State Sodium Batteries with a Polyethylene Glycol Diacrylate–Na_3_Zr_2_Si_2_PO_12_ Composite Electrolyte. Adv. Energy Sustain. Res..

[6] Li H., Xu Z., Yang J., Wang J., Hirano S.I. (2020). Polymer electrolytes for rechargeable lithium metal batteries. Sustain. Energy Fuels.

[7] Sing C.E., Perry S.L. (2020). Recent progress in the science of complex coacervation. Soft Matter.

[8] Timilsena Y.P., Akanbi T.O., Khalid N., Adhikari B., Barrow C.J. (2019). Complex coacervation: Principles, mechanisms and applications in microencapsulation. Int. J. Biol. Macromol..

[9] Arshady R. (1990). Microspheres and microcapsules, a survey of manufactuing techniques Part II: Coacervation. Polym. Eng. Sci..

[10] Umegaki T., Nakayama Y., Kanazawa T. (1976). Thermal Change of Magnesium Highpolyphosphate Coacervates. Bull. Chem. Soc. Jpn..

[11] Umegaki T., Kanazawa T. (1979). Degradation of Magnesium and Calcium Highpolyphosphate Coacervates. Bull. Chem. Soc. Jpn..

[12] de Oliveira L.F.C., Silva M.A.P., Brandão A.R., Stephani R., de Oliveira C.I.R., Gonçalves R.R., Barbosa A.J., Barud H.S., Messaddeq Y., Ribeiro S.J.L. (2009). Amorphous manganese polyphosphates: Preparation, characterization and incorporation of azo dyes. J. Sol-Gel Sci. Technol..

[13] de Oliveira C.I.R., de Oliveira L.F.C., Dias Filho F.A., Messaddeq Y., Ribeiro S.J.L. (2005). Spectroscopic investigation of a new hybrid glass formed by the interaction between croconate ion and calcium polyphosphate. Spectrochim. Acta Part A Mol. Biomol. Spectrosc..

[14] Silva M.A.P., Franco D.F., de Oliveira L.F.C. (2008). New Insight on the Structural Trends of Polyphosphate Coacervation Processes. J. Phys. Chem. A.

[15] Cini N., Ball V. (2014). Polyphosphates as inorganic polyelectrolytes interacting with oppositely charged ions, polymers and deposited on surfaces: Fundamentals and applications. Adv. Colloid Interface Sci..

[16] Momeni A., Filiaggi M.J. (2014). Comprehensive Study of the Chelation and Coacervation of Alkaline Earth Metals in the Presence of Sodium Polyphosphate Solution. Langmuir.

[17] Willot G., Gomez F., Vast P., Andries V., Martines M., Messaddeq Y., Poulain M. (2002). Preparation of zinc sodium polyphosphates glasses from coacervates precursors. Characterisation of the obtained glasses, and their applications. C. R. Chim..

[18] Franco D.F., Barud H.S., Santagneli S., Lamarca R.S., Santos B.F., Silva M.A.P., de Oliveira L.F.C., Ribeiro S.J.L., Nalin M. (2016). Preparation and structural characterization of sodium polyphosphate coacervate as a precursor for optical materials. Mater. Chem. Phys..

[19] Sadeghi R., Jahani F. (2012). Salting-In and Salting-Out of Water-Soluble Polymers in Aqueous Salt Solutions. J. Phys. Chem. B.

[20] Pickup D.M., Newport R.J., Barney E.R., Kim J.Y., Valappil S.P., Knowles J.C. (2014). Characterisation of phosphate coacervates for potential biomedical applications. J. Biomater. Appl..

[21] Kyffin B.A., Foroutan F., Raja F.N.S., Martin R.A., Pickup D.M., Taylor S.E., Carta D. (2019). Antibacterial silver-doped phosphate-based glasses prepared by coacervation. J. Mater. Chem. B.

[22] Franco D.F., Barud H.G.O., Barud H.S., Oliveira Junior O.B., Meneguin A.B., de Oliveira L.F.C., Silva M.A.P., Ribeiro S.J.L., Nalin M. (2020). A review on polyphosphate coacervates—Structural properties and bioapplications. J. Sol-Gel Sci. Technol..

[23] Chen J., Spear S.K., Huddleston J.G., Rogers R.D. (2005). Polyethylene glycol and solutions of polyethylene glycol as green reaction media. Green Chem..

[24] Kim C.W., Someren J.T., Kirshen M., Rha C. (1988). Steric Exclusion of Salts by Polyethylene Glycol. Phys. Chem. Liq..

[25] Kim C.W., Rha C. (2000). Phase Separation of Polyethylene Glycol/Salt Aqueous Two-Phase Systems. Phys. Chem. Liq..

[26] Willauer H.D., Hddleston J.G., Rogers R.D. (2002). Solute Partitioning in Aqueous Biphasic Systems Composed of Polyethylene Glycol and Salt: The Partitioning of Small Neutral Organic Species. Ind. Eng. Chem. Res..

[27] Iqbal M., Tao Y., Zhu Y., Chen D., Wang X., Huang L., Peng D., Sattar A., Shabbir M.A.B., Hussain H.I. (2016). Aqueous two-phase system (ATPS): An overview and advances in its applications. Biol. Proced. Online.

[28] McQueen L., Lai D. (2019). Ionic Liquid Aqueous Two-Phase Systems From a Pharmaceutical Perspective. Front. Chem..

[29] Rodrigues B.P., Limbach R., de Souza G.B., Ebendorff-Heidepriem H., Wondraczek L. (2019). Correlation between ionic mobility and plastic flow events in NaPO_3_-NaCl-Na_2_SO_4_ glasses. Front. Mater..

[30] Wang P., Anderko A. (2001). Computation of dielectric constants of solvent mixtures and electrolyte solutions. Fluid Phase Equilibria.

[31] Christensen J.H., Smith A.J., Reed R.B., Elmore K.L. (1966). Dielectric properties of phosphoric acid solutions at 25 °C. J. Chem. Eng. Data.

[32] Zhuang B., Ramanauskaite G., Koa Z.Y., Wang Z.G. (2021). Like dissolves like: A first-principles theory for predicting liquid miscibility and mixture dielectric constant. Sci. Adv..

[33] Adam O.E.A., Al-Dujaili A.H., Awwad A.M. (2014). Volumetric properites of aqueous solutions of ethylene glycols in the temperature range of 293.15 K–318.15 K. Int. Sch. Res. Not..

[34] Sengwa R.J., Kaur K., Chaudhary R. (2000). Dielectric properties of low molecular weight poly(ethylene glycol)s. Polym. Int..

[35] Chaudhary N., Nain A.K. (2021). Densities, speed of sound, refractive indices, excess and partial molar properties of polythylene glycol 200 + benzyl methacrylate binary mixtures at temperatures from 293.15 K to 318.15 K. J. Mol. Liq..

[36] Shucker R., Gammon R.W. (1970). Raman-scattering selection-rule breaking and the density of states in amorphous materials. Phys. Rev. Lett..

[37] Liland K.H., Kohler A., Afseth N.K. (2016). Model-based pre-processing in Raman spectroscopy of biological samples. J. Raman Spectrosc..

[38] Barkusov E., Macdonald J.R. (2005). Impedance Spectroscopy: Theory, Experiment and Applications.

[39] Almond D.P., Hunter C.C., West A.R. (1984). The extraction of ionic conductivities and hopping rates from a.c. conductivity data. J. Mater. Sci..

[40] Hudgens J.J., Brow R.K., Tallant D.R., Martin S.W. (1998). Raman spectroscopy study of the structure of lithium and sodium ultraphosphate glasses. J. Non-Cryst. Solids.

[41] Koenig J.L., Angood A.C. (1970). Raman spectra of poly(ethylene glycols) in solution. J. Polym. Sci. Part A-2 Polym. Phys..

[42] Samuel A.Z., Umapathy S. (2014). Energy funneling and macromolecular conformational dynamics: A 2D Raman correlation study of PEG melting. Polym. J..

[43] Lattimer R.P. (2000). Mass spectral analysis of low-temperature pyrolysis products from poly(ethylene glycol). J. Anal. Appl. Pyrolysis.

[44] Vrandecic N.S., Erceg M., Jakic M., Klaric I. (2010). Kinetic analysis of thermal degradation of poly(ethylene glycol) and poly(ethylene oxide)s of different molecular weight. Thermochim. Acta.

[45] Han S., Kim C., Kwon D. (1997). Thermal/oxidative degradation and stabilization of polyethylene glycol. Polymer.

[46] de Jager H.J., Prinsloo L.C. (2001). The dehydration of phosphates monitored by DSC/TGA and in situ Raman spectroscopy. Thermochim. Acta.

[47] Marple M.A.T., Avila-Paredes H., Kim S., Sen S. (2018). Atomistic interpretation of the ac-dc crossover frequency in crystalline and glassy ionic conductors. J. Chem. Phys..

[48] Noskov S.Y., Lamoureux G., Roux B. (2005). Molecular dynamics study of hydration in ethanol-water mixtures using a polarizable force field. J. Phys. Chem. B.

[49] Bieze T.W.N., Barnes A.C., Huige C.J.M., Enderby J.E., Leyte J.C. (1994). Distribution of water around poly(ethylene oxide): A neutron diffraction study. J. Phys. Chem..

[50] Shikata T., Takahashi R., Sakamoto A. (2006). Hydration of poly(ethylene oxide)s in aqueous solution as studied by dielectric relaxation measurements. J. Phys. Chem. B.

[51] Kjellander R., Florin E. (1981). Water structure and changes in thermal stability of the system poly(ethylene oxide)-water. J. Chem. Soc. Faraday Trans. 1 Phys. Chem. Condens. Phases.

[52] Guo W., Zhao L., Gao X., Cao Z., Wang Q. (2018). Accurate quantification of hydration number of polyethylene glycol molecules. Chin. Phys. B.

[53] Demos S.G., Raman R.N., Yang S.T., Negres S.A., Schaffers K.I., Henesian M.A. (2011). Measurement of the Raman scattering cross section of the breathing mode in KDP and DKDP crystals. Opt. Express.

[54] Acosta-Maeda T.E., Misra A.K., Porter J.N., Bates D.E., Sharma S.K. (2016). Remote Raman Efficiencies and Cross-Sections of Organic and Inorganic Chemicals. Appl. Spectrosc..

[55] Boschin A., Johansson P. (2015). Characterization of NaX (X: TFSI, FSI)—PEO based solid polymer electrolytes for sodium batteries. Electrochim. Acta.

[56] Garcia-Belmonte G., Bisquert J. (2005). Entropy factor in the hopping frequency for ionic conduction in oxide glasses induced by energetic clustering. J. Chem. Phys..

[57] Exarhos G.J., Miller P.J., Risen W.M. (1974). Interionic vibrations and glass transitions in ionic oxide metaphosphate glasses. J. Chem. Phys..

[58] Palmer R.G., Stein D.L., Abrahams E., Anderson P.W. (1984). Models of Hierarchically Constrained Dynamics for Glassy Relaxation. Phys. Rev. Lett..

[59] Cruje C., Chithrani D.B. (2014). Polyethylene Glycol Density and Length Affects Nanoparticle Uptake by Cancer Cells. J. Nanomed. Res..

[60] Shoar Abouzari M.R., Berkemeier F., Schmitz G., Wilmer D. (2009). On the physical interpretation of constant phase elements. Solid State Ionics.

[61] Hirschorn B., Orazem M.E., Tribollet B., Vivier V., Fratuer I., Musiani M. (2010). Constant-Phase-Element Behavior Caused by Resistivity Distributions in Films: II. Applications. J. Electrochem. Soc..

[62] Anderson O.L., Stuart D.A. (1954). Calculation of Activation Energy of Ionic Conductivity in Silica Glasses by Classical Methods. J. Am. Ceram. Soc..

[63] Nascimento M.L.F. (2007). Test of the Anderson–Stuart model and correlation between free volume and the ‘universal’ conductivity in sodium silicate glasses. J. Mater. Sci..

